# A silent shift? The precarisation of the Dutch rental housing market

**DOI:** 10.1007/s10901-015-9446-5

**Published:** 2015-05-06

**Authors:** Carla Jacqueline Huisman

**Affiliations:** Department of Demography, Faculty of Spatial Sciences, University of Groningen, Groningen, The Netherlands

**Keywords:** Housing, Netherlands, Precarisation, Research agenda, Temporary rent

## Abstract

The traditional Dutch rental contract is permanent (i.e. time unlimited), but there are indications that in recent years the number of temporary rental contracts has increased considerably. Dutch housing policy appears to be responding to this by pursuing deregulation of the conditions under which temporary rent is permitted. It is in this regard startling that there is no reliable data available about the size or character of the temporary sector, and it has thus far not attracted any scholarly attention. Given that temporary rent can be viewed as a form of precarisation, a transfer of risk to citizens, with corresponding negative effects on the lives of those involved, it is imperative to close this knowledge gap. This paper is a first attempt to do this. Firstly, I systematically review the scarce evidence that is currently available, and secondly, I explore why the rise of temporary rent has thus far failed to stimulate any social debate; it appears to constitute a silent precarisation that contrasts with the politically sensitive issue of labour precarisation. In doing so, I will identify the research questions that must be answered if the significance of this process for both tenants and wider welfare-state restructuring is to be fully understood.

## Introduction

‘Everyone is living anti-squat!’ was the title of a recent news item on the Dutch national popular television channel RTL4 (EditieNL, 5 December 2012). Although ‘everyone’ is clearly hyperbole, in the last 15 years, the Netherlands has indeed seen a proliferation of temporary housing arrangements (Buchholz 2009; Van der Molen 2011). It is, for instance, estimated that there are 50,000 anti-squatters; rent paying, live-in security guards occupying empty buildings, in the Netherlands (Renooy 2008). The rise of such precarious housing is striking, given that strong protection of tenants has been the norm in the Netherlands for decades. Normally, rental contracts have unlimited duration and can only be terminated by the landlord for a legally very restricted number of reasons (Lawson 2011). Most rent prices as well as ceilings on annual increases are determined by the government (Haffner and Boumeester 2010, 2014). In contrast, temporary contracts offer hardly any protection to the tenant. They are characterised by their limited and usually unclear duration (Uitermark 2009), while rent prices are often determined by the market.

Officially, to rent out a dwelling with a *temporary contract*, the house owner requires a permit. Permission is restricted to specific situations (i.e. substantial renovation or demolition in the foreseeable future, sale of a previously owner-occupied dwelling) for a limited duration when it is deemed infeasible to rent out dwellings on a permanent basis (Dutch Vacancy law, article 15). The second main form of temporary tenure in the Netherlands is *anti*-*squat*, where the renter functions as a low-level ‘security guard’ for an empty property by living in it. The anti-squatter is not paid for this function and furthermore is expected to pay rent.

Although reliable figures are not available (Priemus 2011), anecdotal evidence suggests that both temporary rent and anti-squat are increasingly becoming normal forms of living. According to an article in the national newspaper *De Volkskrant* (Gualtherie Van Weezel 2012), applications for temporary rent permits doubled in 2011, and for instance, in 2013, almost half of all dwellings rented out newly by Amsterdam housing corporations had a temporary contract (calculated from AFWC 2014:66). Various sources indicate explosive turnover growth in the anti-squat brokerage sector (e.g. Reijmer 2011; Voorn and Heesakkers 2012). Moreover, there is a tendency amongst politicians and policy makers towards expansion of temporary rent. Following an extension of the maximum period of temporary rent from 3 years to 5 years in 2005, the Dutch parliament in 2013 decided on a further relaxation of the regulations to make even longer periods permissible. New, far-reaching changes normalising temporary renting contracts are at the time of writing under discussion in Parliament and expected to come into effect by July 2015.

It is striking, in this regard, that when asked by critical members of parliament how large the temporary rent sector actually is in the Netherlands, the Ministry of Housing responded that no official statistics are available (Report of the Discussion 2012). In academic circles, there has also been very little attention for the issue (but see Priemus 2011). Scientists studying housing tend to focus on topics such as, for instance, the role of real estate capital in the financial crisis of 2007–2008 and its aftermath (e.g. Ronald and Dol 2011) or the economic and social effects of the shift from renting to home ownership (e.g. Helderman et al. 2004). There is, however, little attention for shifts towards temporary contracts inside the rental sector itself. Academic studies of the Dutch housing sector consistently assume that temporary rent is a structurally marginal phenomenon. Although research agendas are of course always socially constructed, this opinion (as discussed later) is at least partly informed by the difficulty of quantifying the size and growth of the sector.

I argue that the absence of rigorous analysis of temporary housing in the Netherlands needs to be addressed because of its potential impact on the wider Dutch housing sector and because of its implications for the more general precarisation of existence in advanced welfare states.

Precarious living arrangements are widely acknowledged to impact negatively upon people’s lives (Cairney and Boyle 2004; Elsinga et al. 2008). Apart from the actual reality of finding oneself without affordable accommodation as the contract ends, the fear of losing one’s home is also influential (Hulse et al. 2011). This influences people’s *ontological security*, a concept coined by Laing (1960) and developed by Giddens (1991, see also Saunders 1990). It refers to the way people give meaning to their life, and how continuity and stability help deal with the experience of everyday events. It is difficult to build a stable life, if it is unclear when one has to move and what opportunities for new accommodation will be available.

The increase in temporary housing arrangements can be seen as a form of precarisation. This concept usually refers to the increase in precarious work: ‘employment that is uncertain, unpredictable, and risky from the point of view from the worker’ (Kalleberg 2009:2). Since the end of the 1970s, due to political and economic restructuring, labour relations have increasingly become characterised by a shift of risks from the employer to the worker (Thompson 2010). As a result, Bourdieu (1998) famously stated that precarity is now everywhere (see also Beck 1992).

It is clear that there are parallels between the socially negative effects of precarious housing and those associated with precarious labour. Yet, there have thus far not been any notable attempts to understand temporary housing in terms of the precarisation literature or to analyse why (in contrast to labour) there is so little critical attention for the growth of temporary housing. I propose a research agenda for analysing the growth of temporary housing through a precarisation lens. This will enable a better understanding of the wider significance of this silent precarisation for broader welfare-state restructuring.

In the remainder of this paper, I begin by formally defining the concept of temporary rental arrangements and describing the main forms that currently exist in the Netherlands. After verifying that there are no meaningful data available regarding the size of the temporary sector, but that there are nevertheless many strong signals that the sector is (rapidly) growing, I then turn to the question of *why* the rise of temporary rent has thus far failed to stimulate any social debate. This silent precarisation is then compared to the politically sensitive issue of labour precarisation. Towards a conclusion, I identify the research questions that must be answered if the significance of this process for both tenants and wider welfare-state restructuring is to be fully understood. Some methodological suggestions for future research are also made.

## Temporary rent in the Netherlands

Before trying to gauge the character and extent of temporary rental arrangements in the Netherlands, it is necessary to first define what is meant exactly by temporary rent. The core of such rental arrangements is that they can be ended without the landlord being legally obliged to give a juridically valid reason for not continuing the lease. This includes fixed-term contracts (e.g. 6 months) where the landlord is under no compulsion to renew the lease after expiration, and unlimited contracts (such as anti-squat contracts) in which no end date is given such that the landlord can terminate the contract at short notice. In addition, I focus on temporary rental arrangements in the context of residence, as opposed to other forms of use, so short-term accommodation for recreation (i.e. holiday homes) or renting for business purposes is excluded from my definition. Lastly, arrangements that do not involve an exchange of money (i.e. letting somebody stay for free in exchange for labour) are not considered.

In the Netherlands, permanent rental contracts have been the norm for a long time. Such contracts are characterised by strong tenant rights, because they have an unlimited duration and can only be terminated by the landlord for a legally restricted number of reasons. The tenant, on the other hand, can in almost all cases always terminate the contract on a short notice of 1 month, without having to supply a reason. Furthermore, the starting level of the rent as well as ceilings on annual increases are in 95 % of all Dutch rental contracts not determined through the market, but subject to regulation (Haffner and Boumeester 2010). Lastly, most housing regulation applies irrespective of the ownership model. That is, rent protection in terms of security of tenure, rent levels and rent increases exists regardless of whether one rents from a private landlord or a non-profit housing corporation.

Permanent contracts in the Netherlands can only be terminated by the landlord for three reasons, which have to be proven in court before eviction becomes legal (Dutch Civil Law Book 7:271–282, Lawson 2011). Firstly, if the tenant fails to meet certain basic legal requirements, a court can terminate the contract. For example, a tenant is obliged to pay the rent, to not cause extreme nuisance (noise, criminal behaviour) and to not damage the property. To secure eviction on such grounds, a landlord has to prove in court that the tenant has persistently violated at least one of these basic requirements. The second legal ground for ending a permanent contract is that a home owner urgently needs the property because he/she or a member of their close family wishes to live in it. In this case, the landlord has to prove that she/he cannot reasonably be expected to seek accommodation elsewhere. (Moreover, if the owner bought the house less than 3 years ago, this ground does not apply.) The last legally allowed reason for terminating a rental contract occurs when the home owner wants to demolish the dwelling or renovate it so extensively that the tenant cannot continue to live in it. In most cases, the landlord is then obliged to supply other, comparable housing to the tenant. Table 1 summarises the main differences between permanent and temporary rental contracts in the Netherlands.

**Table 1:** Comparing permanent rental contracts with temporary rental contracts

Permanent rental contract	Temporary rental contract
Strong tenants’ rights	Weak tenants’ rights
Unlimited duration	Unclear/limited duration
Difficult to terminate	Easy to terminate
Rent regulation	Unregulated

If we now turn to the international context, we note that in Western Europe tenants’ rights are in broad terms quite strong as well. Contracts are either permanent or long term, with a right to renewal. While starting rents are often determined by the market, ceilings on annual increases are often state regulated. In Germany, Denmark and Sweden, for instance, most rental contracts are permanent and subject to largely the same conditions as in the Netherlands (Kemp and Kofner 2010; Scanlon 2011), while, for example, in Belgium, Austria and France, contracts are usually of limited but long duration, and the landlord has to supply a valid reason in court akin to the ones described above for not renewing the contract (Scanlon 2011). In contrast, Anglo-Saxon countries are characterised by weak tenants’ rights and market dynamics (Scanlon 2011). Here, contracts that are either of limited duration without a right to renewal or of unlimited duration but with termination possible at short notice are the norm. In the UK, for instance, leases of 6 months are most common. The situation in the USA, Australia and Canada is similar. The main differences between Anglo-Saxon and European rental systems are summarised in Table 2. The similarity with Table 1 is deliberate: temporary contracts within the otherwise permanent European rental system can be viewed as (emerging) islands of Anglo-Saxon rental norms within that system.

**Table 2:** Comparing European and Anglo-Saxon countries on tenants’ position

European	Anglo-Saxon
Strong tenants’ rights	Weak tenants’ rights
Long/unlimited duration	Unclear/limited duration
Difficult to terminate	Easy to terminate
Rent regulation	Unregulated

The divide between these two rental systems can be seen as a reflection of the difference in welfare-state regimes, originally caused by historically shaped national class alliances (Esping-Andersen 1990). Indeed, whereas Anglo-Saxon countries exhibit traits of liberal forms of government that are minimalistic in provision and rely largely on markets, Western European countries have been characterised as either social democratic or corporatist, resulting in, respectively, universalistic or more hybrid public–private provision, but in both cases with a larger role for government intervention and regulation (Esping-Andersen 1990, 1999). Kemeny (2001) expanded on this thesis. He argues that Anglo-Saxon governments encouraged the market to supply rental housing and supplemented this with a residual public housing stock for a minority of disadvantaged welfare recipients, resulting in a dualistic rental market. In contrast, European countries did not make such a sharp distinction between needs-based state provision of housing and private rental markets, but encouraged competition between profit and non-profit housing provision resulting in unitary rental markets. The last two decades have seen some convergence between the two groups of countries towards more neoliberal policies (Peters 2012), and the Netherlands has been no exception to this trend (Musterd 2014).

Dutch housing regulation is in theory elaborate and strict. The norm is a permanent rental contract, and temporary contracts are only allowed for a limited, restricted number of reasons. Four main forms can be discerned: limited contracts conditional on the status of the house, limited contracts conditional on the status of the renter, unlimited but easy to terminate contracts and unlawful contracts.

Firstly, limited contracts conditional on the status of the *house* are issued when dwellings will be demolished, renovated or sold in the near future and for this reason cannot reasonably be expected to be rented out normally. Over 70 % of all rental housing in the Netherlands is owned by housing corporations, large not for profit organisations (Statistics Netherlands). In the context of urban renewal projects, they often demolish or upgrade entire blocks of buildings. The tenants of those blocks that have permanent contracts usually obtain a right to replacement housing, as well as financial compensation towards moving costs (Huisman 2014). They are given a period of time to choose, and to move into, their replacement housing. Once these tenants leave, the vacated dwellings are rented out on a temporary basis, to prevent them from standing empty. These temporary renters do not obtain the right to rehousing, nor are they recognised as stakeholders in the participatory urban renewal process (Sakizlioglu and Uitermark 2014). Also when a dwelling is empty and for sale, it can be rented out on a temporary contract. In the Netherlands, the sale of a dwelling is *not* a valid reason for the termination of a rental contract. This means that renting out a dwelling with a normal, permanent contract will make it almost impossible for the new owner to evict the tenant and thus make the dwelling uninteresting to buy for a future owner–occupier.

The second form of legal temporary rent is conditional on the status of the *tenant*. In the case of student and youth contracts,^1^ people can rent a dwelling for as long as they fulfil the condition that they are in higher education or below a certain age (Van der Molen 2011). If students graduate or quit their studies, they are granted 6 months to find other housing; if they fail, they can be evicted. The same applies for tenants with so-called youth contracts; if they reach a certain age, they need to vacate their apartment (*Nul20* 12 June 2011 and 6 February 2012). Both forms of conditional contracts are recent phenomena; campus contracts were introduced at the beginning of the 2000s, youth contracts only in 2011, on an experimental basis.

In contrast, contracts with unlimited duration which can be terminated by the landlord at short notice without reason are forbidden in the Netherlands. This is why anti-squat agencies maintain that they do not rent out dwellings, but instead employ security guards to prevent squatting and vandalism.^2^ These ‘security guards’, however, do not receive a salary for their services, but have to pay a considerable fee for the privilege of guarding a building, a fee that often reaches the level of normal rents in the Netherlands (Dutch Union of Tenants 2014). Their contracts are notoriously precarious; it is usual that the landlord is permitted to terminate the contract with only 2 weeks’ notice (Martínez-López 2013).^3^ As such, anti-squat is a deliberate attempt to circumvent strict Dutch housing regulation. Anti-squat agency Alvast, for instance, makes this clear on its website: ‘The contracts of Alvast are formulated in such a way that the temporary users cannot claim rights pertaining to protection of tenure’.^4^ However, in some of the few cases that have been legally tested, the courts have ruled that the ‘guards’ should be considered renters with normal, permanent rental contracts (e.g. Amsterdam Court of Justice 2011). But given the continuing shortage of affordable housing in the Netherlands, many anti-squatters are happy to have secured some form of housing and do not dare or want to claim their legal rights.

The last form of temporary housing consists of the grey market. Given the scarcity of housing in some parts of the Netherlands, many landlords manage to impose conditions on their tenants that are not legal, such as a contract for a limited period of time or an unlimited contract with the possibility of short-notice termination as well as rent levels or increases above what is allowed by law. The practice whereby renters rent out their dwellings to somebody else without permission of the landlord also falls in this category. Both anti-squat and the grey market reflect that security of tenure for tenants is not only just determined by existing legislation, but also on circumstances in practice, as well as how tenants experience that reality (Hulse and Milligan 2014).

## No data, but likely growing

Having characterised the four main forms of temporary rent in the Netherlands, it is relevant to ask how often they occur. Unfortunately, there are currently no meaningful statistics available. At the national aggregate level, nothing is known. The state-commissioned National Survey on Housing in the Netherlands (WoON), for example, that is repeated every 3 years and for which more than 40,000 respondents are interviewed, does not include any question about the form of rental contracts. Statistics Netherlands does not have any data either. An isolated housing corporation or municipality might, from time to time, publish some data on temporary rent, but this occurs in a purely ad hoc fashion and is an inadequate basis for systematic, structured analysis.

Data on the different forms of temporary rent are also sparse or non-existent. For example, the only estimate we have of the number of anti-squatters in the Netherlands (between 20,000 and 50,000 on a total population of 16.4 million) comes from Renooy (2008:36). His sources are one interview with an unidentified respondent and the text found on the website of one anti-squat agency (Renooy 2008). Because of the lack of data, the upper bound of his estimate is continually repeated in the media (e.g. Van der Tol 2011; Van der Ploeg 2012). Anti-squat agencies are generally reluctant to release detailed data about their activities, arguing that it could undermine their position in the market.

Most striking, however, is the lack of structured data regarding the more official forms of temporary rent. As far as we have been able to ascertain there are no pooled statistics available at any level regarding the number of (formerly owner occupied) dwellings that are being temporarily rented out while they are being sold. Similarly, there is little data available on the number of student/youth contracts. Furthermore, temporary rent in the context of urban renewal is not monitored. The Dutch federation of housing corporations (Aedes) collects no data on temporary rent (personal communication Aedes, 5 March 2015). Yet, there are several reasons for thinking the temporary sector is growing.

One important reason is the impact of the global financial crisis of 2007–2008 and the ensuing global economic recession. Many urban renewal projects have been delayed or cancelled. Even before the crisis, many housing corporations already had the tendency to switch from permanent to temporary rent many years before the urban renewal event nominally justifying the switch—renovation or demolition—actually takes place (de Zeeuw 2005, 2010). In this way, the housing corporation can avoid displacing permanent renters later in the trajectory and hence avoid the need to rehouse and compensate them. The price, of course, is that the dwelling potentially spends many years more than necessary (or, indeed, legally allowed) outside the regular, permanent rental circuit. The crisis has only served to exacerbate the phenomenon: planned dates for renovation and demolition are postponed, but the temporary rent persists. The financial crisis has also impacted on the private market. The number of dwellings for sale has been continually increasing, while the average time that a dwelling is for sale has significantly increased (NVM 2013). The crisis has particularly impacted on office buildings, fuelling a boom in anti-squat agencies’ turnover (Voorn and Heesakkers 2012).

While the phenomena described above are market driven, in the sense that lack of access to investment capital facilitates a lengthening of a temporary phase in the life of a building, they have been accompanied by regulatory shifts that began before the crisis but which have accelerated in its wake. Until 2005, temporary rent was only allowed in the case of advanced plans for demolition/far-reaching renovation or a vacated, previously owner-occupied home being for sale. In such cases, a permit could be obtained for renting the building out temporarily for a year. Afterwards, the permit could be renewed each year, up to a total of 3 years. In 2005, this period was extended to 5 years. After a successful lobby of, amongst others, the Dutch association of owner–occupiers (Vereniging Eigen Huis), the Dutch Parliament decided in 2013 to relax the rules even further. Private home owners selling their old dwelling on the market are no longer obliged to seek renewal of their temporary rent permit, but immediately obtain permission for temporary rent for 5 years, and in such cases, all regulations on the maximum rent level have been lifted. The argument is that many home owners did not intent to become landlords and that they struggle to pay the mortgage on the low regulated rents. Housing corporations are now permitted to temporarily rent out their houses for up to 7 years, while office space can be rented out for living purposes for up to 10 years. Note that this does not mean that the tenant receives a single contract for 10 years. Rather, the tenant has a series of shorter contracts, often between 6 months and 2 years, which the house owner renews repeatedly. The lengthening of the legally allowed period of temporary rent up to 7 or 10 years inevitably raises the question how temporary temporary rent actually is.

While the consequences of these latest changes of regulations were yet unknown because they were just coming into effect in the summer of 2013, at that very moment, a campaign for further relaxation begun. Kick-started by Amsterdam housing corporations, notably a publication by corporation Stadgenoot (de Langen and Anderiesen 2013), this lobby soon included the national association of housing corporations (Aedes), the aldermen in charge of housing of the four major cities Randstad, as well as the small orthodox Christian political party ChristenUnie (Nolles 2013; Schouten et al. 2013). Their arguments of creating living space for currently underserved target groups such as youngsters and increasing residential mobility convinced the current Minister of Housing Stef Blok. In summer 2014, he proposed far-reaching changes to the law. Temporary renting contracts will be introduced as a normal form of tenure. The only restrictions will be that the contract can be for a maximum of 2 years and that it cannot be extended for the same tenant. However, the modest amount of attention this proposal drew concerned *not* this previously unheard of major abolishment of rent protection, but focused instead exclusively on marginal specific measures for certain target groups such as students, youngsters or problematic renters (but see Dutch Bar Association 2014). Indeed, Minister Blok (2014) downplayed the impact of these changes arguing that because of the transaction costs involved, landlords will not engage en masse in temporary contracts. The proposal was received positively by the majority of Parliament, and at the time of writing, it is expected that the changes to the law will come through by 1 July 2015.

As such, it is entirely plausible that we will observe a further expansion of temporary living arrangements in the near future. In the previously highly regulated housing market of the Netherlands, this will constitute a significant shift towards weaker tenants’ rights. Yet, this change in the distribution of rights and risks between landlords and tenants has not sparked any discussion in the public domain so far. In the next section, I develop a tentative exploration why this shift is has been silent so far, and how we can understand it.

## A silent shift

No structural research documents the extent of temporary rental arrangements in the Netherlands, while there are ample signals that it is increasing. I identify four principal reasons for this lack of non-anecdotal, non-incidental studies. The first explanation lies in the assumption that temporary rent adds to the stock, and the second in the assumption that it constitutes a transient phase in people’s lives. The third cause concerns the use of temporary rent to ‘patch up’ certain problems in housing policy, without considering the aggregate effect of many such patches. The fourth factor is connected to the inherent difficulty of measuring the phenomenon of temporary rent.

As regards the first point, a common assumption amongst politicians, policy makers and researchers in the Netherlands, is that temporary rent is *adding* to the current housing stock, because the dwellings concerned otherwise would have remained empty. Given the continuing scarcity of housing in (some parts of) the Netherlands, adding more housing to the stock is conceived as a positive development. However, given the growth of temporary rent, the question is whether rather than adding, it is replacing other, more structural uses of the housing stock, especially given the continuously lengthening periods of time involved. In the case of home owners struggling to sell their old home, the bonus now put on temporary rent by the recent decision of the Dutch government to significantly deregulate it will almost certainly lead to replacement; as long as the dwelling is at least nominally kept on the market, a landlord will naturally opt for a temporary contract of fixed duration with a free-market rent above a permanent contract that is difficult to terminate with a regulated rent. In the context of urban renewal, a dwelling that is rented out temporarily for 5–10 years as it waits for a renovation that might never happen should also be considered to be *replacing* a dwelling that could have been rented out permanently.

The second common assumption explaining the disinterest in temporary rent in the Netherlands that prevails amidst the Dutch housing policy community concerns the renters themselves. It is often assumed they are young, highly educated, unattached and thus robust to the insecurity of temporary rent. The precarious living situation will only constitute a short and *transient* phase in their lives, it is thought, after which they will move on more secure forms of housing, such as a permanent rental contract or owner-occupied housing: the stereotypical temporary renter is a student. But there is no reason to assume that other, less advantaged people will *not* end up in such insecure housing. The continuing scarcity of affordable housing in many Dutch cities, the increasing shift towards a market-based model of housing, and the lack of any safety net for outsiders and newcomers imply that pressing need, rather than free choice, is often a determinant of how and where people live (Dutch Union of Tenants 2013). The extension of the maximum permissible period of temporary rent to 10 years, and the corresponding emergence of almost ‘permanent temporariness’, also means it is more likely that initially ‘robust’ temporary renters will lose this robustness during the tenancy, i.e. they will shift into a new life phase such as family formation.

The third factor behind the lack of interest in temporary rent is the fact that most forms of temporary tenure are usually only considered from the angle of the specific problem they are supposed to be address. Policy makers, politicians and academics focus on distinct problems, such as keeping houses occupied during urban renewal or relieving home owners with financial problems due to double mortgages because they bought a new home without having sold the old one. The compound effects of all these isolated policy responses are seldom considered, leading to the assumption that temporary rent arrangements are a marginal phenomenon. However, I argue that if we take the extent of and increase in the various forms of temporary rent arrangements as a whole, this constitutes a significant shift towards the temporary.

The above-described assumptions that temporary renting is a positive, though marginal phenomenon because it adds housing to the stock that is used as a temporary stop-over by people able to deal with the insecurity, combined with a focus on individual policy responses explain why there is no interest of researchers, politicians, policy makers and other housing professionals in collecting data. But another reason is that collecting data on the character and extent of temporary rent presents a daunting methodological challenge. The gold standard approach to find out how many people live in temporary housing in the Netherlands would be, of course, to count them or estimate their number through a random sample. Unfortunately, due to the specific nature of temporary housing, this will be difficult. The semi-legal and fleeting character of precarious living does not lend itself very well to random sampling techniques. For instance, drawing a sample from the municipal population register (GBA) will omit many people. Although legally people are obliged to register at the address they factually live (Law on the Municipal Population Register articles 65–66), many municipalities do not allow people to register in buildings not designated for living. As a result, many anti-squatted buildings are officially registered as empty. Secondly, in the case of dwellings, home owners often do not allow temporary renters to register (see for an example of a housing corporation Central Council of Appeal 2008). Temporary renters are furthermore unlikely to respond to general surveys because of their semi-legal and precarious living status. Dutch survey response rates are low at 55 % at best (Stoop 2005), and the non-response is heavily biased exactly towards renters and low-income groups (Te Riele 2002). Furthermore, as mentioned earlier, anti-squat agencies have been shown to be reluctant to divulge information.

In conclusion, I argue that the lack of research and the related lack of data have meant that an important shift in the Dutch rental system has gone unobserved. The Netherlands seems to be shifting towards the Anglo-Saxon model of weaker tenant rights, without this being an explicit policy goal. This stands in stark contrast with the shift towards home ownership, which has been extensively promoted by the Dutch government and extensively studied. Moreover, it also contrasts with the elaborate attention devoted to a similar, earlier shift in another domain, namely the demise of the permanent labour contract in the context of the rise of temporary contracts.

Interestingly, this shift seems to be more important for its symbolic meaning than for its statistical importance: between 1992 and 2012, the number of Dutch workers with a permanent contract decreased only from 75 to 69 %, while the number of temporary employed workers increased from 13 to 16 %, and the number of self-employed persons increased from 12 to 15 % (Statistics Netherlands, see also Cörvers et al. 2011). However, it would be wrong to conclude that the discussions of the last 15 years about the precarisation of labour are *much ado about nothing*. On the contrary, the debate about this shift of risks from employer to worker (Kalleberg 2009) is at the heart of (European) welfare-state reform and the underwhelming statistics potentially mask a generational trend. Could, for example, the rise of the temporary labour contract be an age-related phenomenon? That is, will the youngsters that now have temporary contracts obtain a permanent contract later on in their career or will they have to spend most of their professional lives switching from one temporary contract to another?

Analogous questions could be posed regarding the emergence of temporary rent. How large is the sector? How is it composed? What is its wider significance for welfare-state reform? Is the extent of the shift masked by generational factors? Yet, as observed earlier in this article, there is neither data nor debate. Potentially, then, the sector is being precarised without any of the critical attention associated with labour precarisation. This silent shift therefore deserves attention from researchers, both at the theoretical and at the methodological level.

## Research agenda: investigating the precarisation of the Dutch housing market

To fill the gap in our knowledge, we need to know more about the various forms of temporary rental arrangements in the Netherlands. Are there more contracts with a limited length, or are there more contracts that run indefinitely but can be ended with 2 weeks’ notice? What are the main categories of people in precarious housing? Are they indeed young and unattached, as often is assumed, or are there also more vulnerable people ending up in temporary rent? Are motivations for being in this form of tenure mainly positive (low cost, easy access), negative (no alternative) or neutral? What combinations of such push and pull factors occur? How do residents experience this form of housing, is it stressful, problematic, adventurous? Is temporary rent geographically concentrated in the four largest cities known as the Randstad, or in urban areas, or is it occurring throughout the country? And what proportion of the Dutch housing market is non-permanent? These are some of the questions that need to be answered if we want to seriously engage with this neglected form of housing. But apart from these empirical questions into what, who and how many, there are also pressing broader questions. What does this shift signify? What is the history? Who are the main actors involved? Can we observe similar changes in other countries, in Europe perhaps? How is the discourse pertaining to living in insecure circumstances evolving over time? What can we learn from the labour precarisation literature, and what are the differences and similarities? I believe these questions (organised in Table 3) can be summarised as follows:

**Table 3:** Researching the silent shift: topics and methods

Main research topics	Examples of relevant dimensions	Quantitative methods	Qualitative methods
Forms of temporary rental arrangements (what) and volume (how much)	Period of contract Termination of contract Rationale for temporary contract Legal status of contract Rights according to contract Responsibilities according to contract Rent price levels and increases Number of contracts Number of households and individuals	*Primary data collection* Surveys (cross-sectional + panel): General surveys Targeted surveys *Secondary data analysis* Statistical data collected by Associations of house owners Tenants advocacy organisations Government Research bureaus Indirect data Permits issued Turnover statistics GIS	*Primary data collection* In-depth interview/focus groups Tenants Other stakeholders Ethnography *Secondary data analysis* Document analysis Media Policy Contracts Laws
History/growth over time (when)	Recent years Recent decades Pre-/postwar period Changes in regulation		
People involved with precarious housing (who)	Renters Landlords of different scales and with different organisation forms Government (policy makers and politicians) on different levels; municipalities, central government Advocacy organisations/lobby groups Media		
Motivations for being in precarious housing (why)	Interaction of push and pull factors; positive (low cost, easy access)/negative (no alternative)/neutral		
Residents’ experiences (how)	Perception of their housing situation Impact on other parts of their lives Moment in life course		
Physical and geographical pattern (where)	Residential dwellings/business spaces Physical state of buildings Urban/rural spread/concentration Distribution within cities/regions Situation in neighbouring countries/Europe/outside Europe

- What is the extent and character of precarious housing in the Netherlands?
- How should we understand the silent growth of temporary rent in the Netherlands in the context of precarisation?

Concerning method, it would be a great improvement if large-scale housing surveys (such as WoON) would recognise temporary rent as an existing form of housing and if questions concerning this form of tenure would be incorporated. In this way, the empirical foundations for accurately monitoring the character as well as the growth of the sector *over time* can be laid. Alternatively, data could be collected by devising and administering surveys targeted specifically on temporary rent. To obtain a deep understanding of how insecure housing impacts on people’s lives, more qualitative methods can be employed. In particular, interviews with tenants will yield more insight into the experiences of people. The more general shift can be investigated by studying policy documents, media content, as well as through interviewing key figures such as directors of anti-squat agencies, housing corporations and politicians. These are just a few examples of how one could go about closing the gap in our knowledge. Undoubtedly, other, more creative ideas could be employed. But my main point is not *how* we should research precarious housing in the Netherlands, but *that* the issue urgently requires attention.

A practical goal of proposing this research agenda is to provide policy makers with data on temporary housing in the Netherlands. This is especially relevant given that there is a clear trend in policy towards further expansion of the temporary sector. By more accurately quantifying the size and character of the sector, we can clarify how far existing policy actually reflects, and influences, the reality of temporary rent on the ground. In this way, the desirability of future expansion of the sector could be critically assessed. Indeed, perhaps the most important goal of proposing this research agenda is to stimulate an open and fundamental societal debate about the appropriate balance between legal protection of renters and the rights of landlords. By gathering and making public information about the extent and the character of precarious housing in the Netherlands, not only a much needed empirical basis for such discussions will be created, but also the findings themselves will help put the topic of this silent shift on the agenda.

## Footnotes

1 These contracts are commonly referred to in Dutch as *campus* contracts; however, most of the dwellings involved are not situated on a university campus, but in ordinary non-university neighbourhoods.

2 Anti-squat started off in the 1990s as a way to prevent squatting, but has turned into a form of tenure in its own right, while the Dutch squatting movement has been marginalised (van Gemert et al. 2009).

3 Furthermore, contracts often include far-reaching restrictions: “Anti-squat regulations comprise: no parties, no children, no pets, no smoking, no guests overnight, no vacation (permission required to leave the house for more than three days), maintenance of house and garden, no contact to the press.” (Buchholz 2009:215, see also Heijkamp 2009).

4 My translation from Dutch, see www.alvast.nl/eigenaren/leegstands-beheer-anti-kraak/, last visited 15 March 2015.
